# Graphene Oxide Nanoparticle–Loaded Ginsenoside Rg3 Improves Photodynamic Therapy in Inhibiting Malignant Progression and Stemness of Osteosarcoma

**DOI:** 10.3389/fmolb.2021.663089

**Published:** 2021-04-22

**Authors:** Shou-Liang Lu, Yan-Hua Wang, Guang-Fei Liu, Lu Wang, Yong Li, Zhi-Yuan Guo, Cai Cheng

**Affiliations:** ^1^No. 1 Orthopedics Department, Cangzhou Central Hospital, Cangzhou, China; ^2^ECG Examination Department, Cangzhou Central Hospital, Cangzhou, China

**Keywords:** osteosarcoma, graphene oxide, photodynamic therapy, ginsenoside Rg3, stemness

## Abstract

Osteosarcoma serves as a prevalent bone cancer with a high metastasis and common drug resistance, resulting in poor prognosis and high mortality. Photodynamic therapy (PDT) is a patient-specific and non-invasive tumor therapy. Nanoparticles, like graphene oxide have been widely used in drug delivery and PDT. Ginsenoside Rg3 is a principal ginseng component and has presented significant anti-cancer activities. Here, we constructed the nanoparticles using GO linked with photosensitizer (PS) indocyanine green (ICG), folic acid, and polyethylene glycol (PEG), and loaded with Rg3 (PEG–GO–FA/ICG–Rg3). We aimed to explore the effect of PEG–GO–FA/ICG–Rg3 combined with PDT for the treatment of osteosarcoma. Significantly, we found that Rg3 repressed proliferation, invasion, and migration, and enhanced apoptosis and autophagy of osteosarcoma cells, while the PEG–GO–FA/ICG–Rg3 presented a higher activity, in which NIR laser co-treatment could remarkably increase the effect of PEG–GO–FA/ICG–Rg3. Meanwhile, stemness of osteosarcoma cell–derived cancer stem cells was inhibited by Rg3 and PEG–GO–FA/ICG–Rg3, and the combination of PEG–GO–FA/ICG–Rg3 with NIR laser further significantly attenuated this phenotype in the system. Moreover, NIR laser notably improved the inhibitor effect of PEG–GO–FA/ICG–Rg3 on the tumor growth of osteosarcoma cells *in vivo.* Consequently, we concluded that PEG–GO–FA/ICG–Rg3 improved PDT in inhibiting malignant progression and stemness of osteosarcoma cell. Our finding provides a promising and practical therapeutic strategy for the combined treatment of osteosarcoma.

## Introduction

Osteosarcoma is the prevalent malignant bone cancer principally befalling throughout adolescence and childhood (Du et al., 2021; Gioti et al., 2021). Osteosarcoma emphasizes the profoundly malignant phenotypes, and 75% of osteosarcoma cells migrate nearby tissues (Li et al., 2021; Rathore et al., 2021). Although the overall 5-years survival incidence of osteosarcoma patients has improved by applying surgery and chemotherapy over the past 30 years, prognosis continues unsatisfactory due to drug resistance and metastasis (Wen et al., 2020; Tornin et al., 2021). Therefore, it is urgently needed to develop innovative therapeutic strategies for osteosarcoma (Zheng et al., 2021).

Graphene serves as a two-dimensional sp2-based carbon nanosheet of the honeycomb lattice and presents extraordinary physicochemical features for broad utilization, including biomedicine, nanocomposite materials, energy conversion/storage, and nanoelectronics (Ahamed et al., 2021; Pendergast et al., 2021). Graphene oxide (Tang et al., 2018) is a graphene derivative and is widely applied for photothermal therapy, biological imaging, and drug delivery (Lin et al., 2018; Marrella et al., 2018; Krasteva et al., 2019). Photodynamic therapy (PDT) is a patient-specific and non-invasive tumor therapy by absorbing and transferring energy using a photosensitizer (PS), generating singlet toxic oxygen (Tian et al., 2011; Liu et al., 2018; Gautam et al., 2020). GO has been used in the combination of PDT and PPT for cancer treatment (Li et al., 2015; Sun et al., 2018). Indocyanine green (ICG) is a tricarbocyanine/amphiphilic dye approved by the United States Food and Drug Administration for the application of biomedicine (Ocsoy et al., 2016; Akbari et al., 2017). ICG presents several advanced properties, such as low toxicity, strong emission band (800–820 nm), and effective absorption band (780 nm), which make ICG widely optimal for the application in animals, tissues, and cells (Sharker et al., 2015). Folic acid (Ahmmed et al., 2019), as a targeted agent for cancer cells, has been conjugated with GO by the imide linkage in the treatment of cancers (Huang et al., 2011; Hu et al., 2013). A recent study showed that polyethylene glycol (PEG)–GO–FA/ICG nanoparticle-delivered MutT homolog 1 inhibitor improves the chemo-photodynamic therapy in osteosarcoma (Huang et al., 2020). Ginsenoside Rg3 serves as a primary ginseng component and presents angiogenesis inhibitory potential in conventional biomedicine (Ahmmed et al., 2019). Rg3 exhibits anti-tumor properties in various cancer models, including lung cancer, breast cancer, and ovarian cancer (Sun et al., 2017; Song et al., 2020). Rg3 has been found to induce apoptosis and repress migration of cancer stem cells (Tang et al., 2018). However, the nanoparticle-based delivery system and its role in the combination of chemo-photodynamic therapy for osteosarcoma are still unclear.

In this study, we constructed a PEG–GO–FA/ICG nanoparticle loaded with Rg3 (PEG–GO–FA/ICG–Rg3) and identified that the co-treatment of PEG–GO–FA/ICG–Rg3 and PDT significantly inhibit the malignant phenotypes of osteosarcoma *in vitro* and *in vivo.*

## Materials and Methods

### Nanoparticle Preparation

Indocyanine green, FA, and PEG were obtained (Sigma, United States) and graphene oxide was made by graphite powder using Hummers and Offman’s method, with small modifications according to the previous description (Anirudhan et al., 2020). GO (2 mg/ml) and diamino-PEG were used to construct the PEG–GO using acetylation reaction. FA molecule and ICG were further conjugated to the PEG–GO through carbodiimide-mediated covalent bond formation as the previous reports (Huang et al., 2020). The PEG–GO–FA/ICG–Rg3 was prepared using Rg3 (1–10 mg) and PEG–GO–FA/ICG 0.1 mg in the dark solution and stirred for 24 h at 4°C. The drug loading content was calculated: loading content = loaded drug (mg)/PEG–GO–FA/ICG (mg). The nanoparticles were observed by transmission electron microscopy (TEM; JEOL, Japan) and scanning electron microscope (SEM; HITACHI, Japan). The nanoparticles were characterized by atomic force microscopy (AFM).

### Drug Release Analysis

The dialysis bag method was applied to assess the drug release *in vitro* (Anirudhan et al., 2020). The nanoparticle solution was dispersed with 1 ml of PBS (pH 7.4 or pH 5.0) containing 0.1% Tween 80, placed into a dialysis bag, and placed in a centrifuge tube containing 30 ml of the corresponding release medium. The *in vitro* release test was performed on a 37°C shaker with 100 rpm. Release medium (200 μl) was taken at 0.5, 1, 2, 3, 4, 6, 8, 12, 24, and 48 h, and an equal volume of fresh release medium was added. HPLC was used to determine the drug concentration in the released sample and calculate the cumulative release.

### Cell Culture and Treatment

The MG63 and U2OS cells were maintained in the laboratory and were cultured at 37°C with 5% CO_2_ in DMEM (GE, United States) containing FBS (10%; Gibco, United States), streptomycin (0.1 mg/ml, Gibco, United States), and penicillin (100 U/ml; Gibco, United States). To evaluate the effect of nanoparticles on PDT, the cells were treated with NIR laser (808 nm; SFOLT, China) for 120 s.

### CCK-8 Assays

CCK-8 assays detected the MG63 and U2OS cell viabilities. About 2 × 10^4^ cells were put into 96 wells and cultured for 12 h. Then the cells were used for the treatment. After 72 h, the cells were added with a CCK-8 solution (KeyGEN Biotech, China) and cultured for another 2 h at 37°C. The ELISA browser was applied to analyze the absorbance at 450 nm (Bio-Tek EL 800, United States).

### Colony Formation Assays

Around 1 × 10^3^ MG63 and U2OS cells were placed into six-well plates and incubated in DMEM at 37°C. After 14 days, the MG63 and U2OS cells were washed using PBS buffer, added with methanol for 30 min, and stained with crystal violet dye at the dose of 1%. The colony formation numbers were calculated.

### Transwell Assays

Transwell assays analyzed the cell migration and invasion of MG63 and U2OS cells by applying a Transwell plate (Corning, United States) according to the manufacturer’s guidance. The upper chamber was plated with 1 × 10^5^ MG63 and U2OS cells and the bottom chamber was added with DMEM with 10% FBS, solidified using paraformaldehyde (4%), and then dyed using crystal violet. Invaded and migrated cells were recorded and calculated.

### ROS Production Analysis

The cellular ROS production was analyzed using 7’-dichlorodihydrofluorescein diacetate (DCFH-DA) staining (Sigma-Aldrich, United States) according to the manufacturer’s instruction. Briefly, about 1 × 10^4^ SH-SY5Y cells were plated on 96-well black dishes in the standard culture medium and were cultured overnight. Cells were stained with DCFH-DA (100 μM), and the fluorescence intensity of different groups was analyzed by a fluorescent reader by 540 nm emission wavelength and 480 nm excitation wavelength.

### Analysis of Cell Apoptosis

About 2 × 10^5^ MG63 and U2OS cells were plated on six-well dishes. Cell apoptosis was assessed by employing the Annexin V-FITC Apoptosis Detection Kit (CST, United States) using the manufacturer’s instruction. Shortly, about 2 × 10^5^ washed cells were collected by binding buffer and were dyed at 25°C, followed by the flow cytometry analysis.

### Analysis of CD117- and Stro-1-Positive Cells

The CD117- or Stro-1-positive osteosarcoma cells were analyzed using flow cytometry analysis as the previous report (Zhang et al., 2018). About 1 × 10^6^ cells were resuspended in 150 ml Dulbecco’s Hanks Balanced Salt Solution with CD117 (CST, United States) or Stro-1 (CST, United States) antibodies, followed by culture for 30 min and flow cytometry analysis.

### Sphere Formation Assays

The cancer stem cell properties were analyzed by sphere formation assays. About 1 × 10^4^ CNE2 and S26 cells were plated into 24-well plates and cultured for 5 days. After that, the tumor sphere formation was captured, and the images were analyzed by using a fluorescence microscope.

### Western Blot Analysis

Total proteins were isolated from the cells by using RIPA (CST, United States) and the quantification was performed by BCA Protein Quantification Kit (Abbkine, United States). Equal protein samples in SDS–PAGE were transferred to PVDF membranes (Millipore, United States) and incubated in 5% milk at 25°C for 2 h and the primary antibodies at 4°C overnight. The second antibodies (Abcam, United States) were applied to hatch the membranes for 1 h at 25°C, followed by the visualization using ECL Kit (Beyotime, China). The primary antibodies applied in this study comprised Bcl-2 (Abcam, United States), Bax (Abcam, United States), caspase3 (Abcam, United States), cleaved caspase3 (Abcam, United States), LC3B (Abcam, United States), beclin-1 (Abcam, United States), p62 (Abcam, United States), Sox2 (Abcam, United States), Oct3/4 (Abcam, United States), Nanog (Abcam, United States), and β-actin (Abcam, United States).

### Analysis of Tumorigenicity in Nude Mice

The tumor growth of nasopharyngeal carcinoma cells *in vivo* was analyzed in nude mice of Balb/c (male, 4 weeks old) (*n* = 5). About 1 × 10^7^ MEG3 cells were subcutaneously injected into the mice. After 3 days of injection, we measured tumor growth every 3 days. We sacrificed the mice after 21 days of injection, and tumors were scaled. Tumor volume (V) was observed by estimating the length and width with calipers and measured with the method × 0.5. Animal care and method procedure were authorized by the Animal Ethics Committee of Cangzhou Central Hospital.

### Statistical Analysis

Data were expressed as mean ± SD, and the statistical analysis was conducted using GraphPad Prism 7. Unpaired Student’s *t*-test was used to compare two groups. *P* values < 0.05 were considered as statistically significant.

## Results

### The Characterization of PEG–GO–FA/ICG–Rg3

**FIGURE 1:**
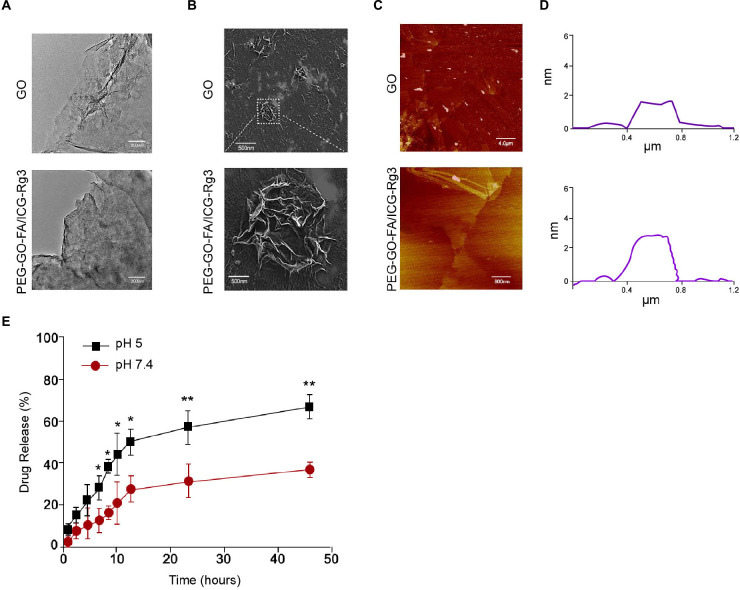
The characterization of PEG–GO–FA/ICG–Rg3. **(A)** The nanoparticle morphology observation using TEM. **(B)** The dispersibility and morphology observation of nanoparticles using SEM. **(C,D)** The nanoparticle thickness observation using AFM. **(E)** The drug release analysis of PEG–GO–FA/ICG–Rg3 at pH 5.0 and pH 7.4.

The PEG–GO–FA/ICG was successfully constructed, in which ICG was the PS, FA was the active targeting agent, and PEG was the linking agent. We observed the morphology using the TEM and SEM (Figures 1A,B). The particle morphology was evaluated by AFM and the thickness was 1.85–3.11 nm (Figures 1C,D). The drug release analysis demonstrated that the PEG–GO–FA/ICG–Rg3 effectively released the drug at pH 5.0 and pH 7.4 (Figure 1E).

### The Effect of PEG–GO–FA/ICG–Rg3 on Proliferation, Invasion, and Migration of Osteosarcoma Cells

**FIGURE 2:**
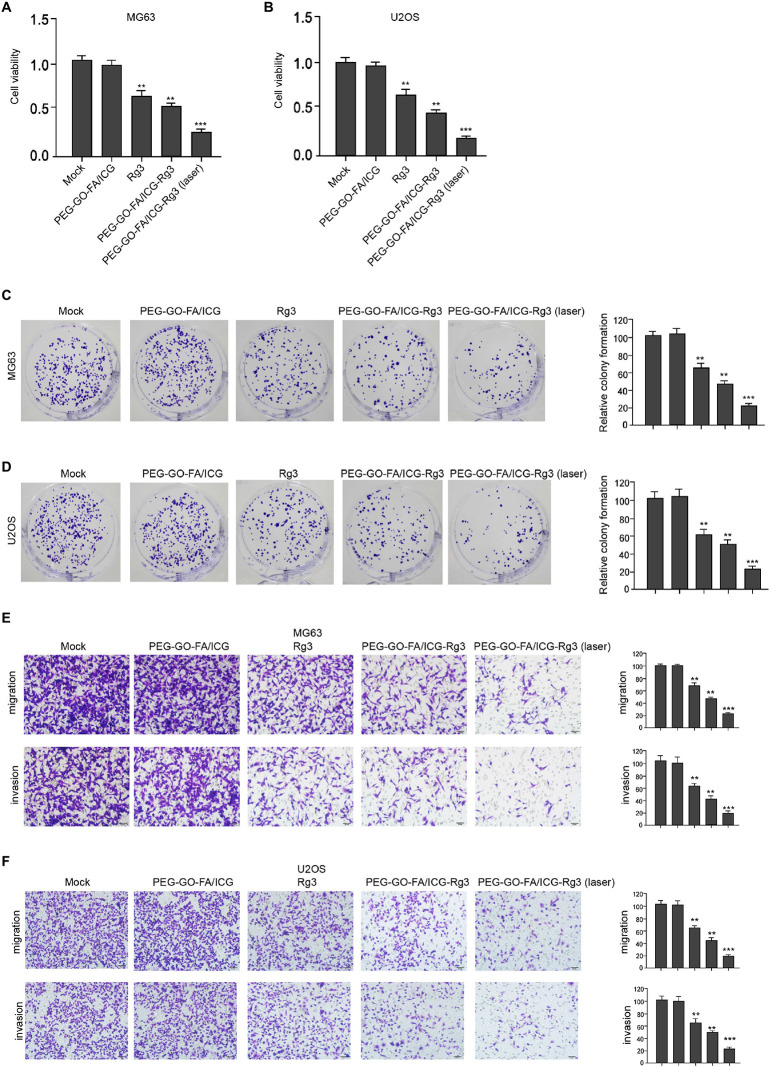
The effect of PEG–GO–FA/ICG–Rg3 on proliferation, invasion, and migration of osteosarcoma cells. **(A–D)** The MG63 and U2OS cells were treated with PEG–GO–FA/ICG, Rg3, and PEG–GO–FA/ICG–Rg3, or co-treated with PEG–GO–FA/ICG–Rg3 and NIR laser. **(A,B)** CCK-8 analysis in the cells. **(C,D)** Colony formation assays in the cells. **(E,F)** Transwell analysis in the cells. Mean ± SD, **P < 0.01, ***P < 0.001.

The effect of the nanoparticles on the proliferation, invasion, and migration in osteosarcoma cells was analyzed. Remarkably, compared with the mock and PEG–GO–FA/ICG treatment group, Rg3 treatment reduced the cell viability in the MG63 and U2OS cells and PEG–GO–FA/ICG–Rg3 showed a higher effect (Figures 2A,B). Importantly, the co-treatment of PEG–GO–FA/ICG–Rg3 and NIR laser significantly stimulated the inhibition of viability of MG63 and U2OS cells in the system (Figures 2A,B). As expected, colony formation assays confirmed that NIR laser enhanced the PEG–GO–FA/ICG–Rg3-repressedcolony formation of MG63 and U2OS cells (Figures 2C,D). Moreover, the invasion and migration were suppressed by Rg3 and PEG–GO–FA/ICG–Rg3, in which the co-treatment of NIR laser notably improved the inhibitory effect of PEG–GO–FA/ICG–Rg3 on the invasion and migration of MG63 and U2OS cells (Figures 2E,F).

### The Effect of PEG–GO–FA/ICG–Rg3 on Apoptosis of Osteosarcoma Cells

**FIGURE 3:**
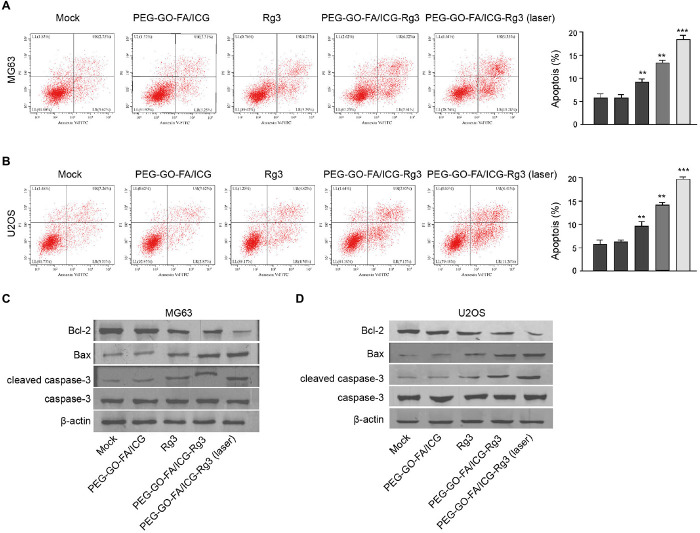
The effect of PEG–GO–FA/ICG–Rg3 on apoptosis of osteosarcoma cells. **(A–D)** The MG63 and U2OS cells were treated PEG–GO–FA/ICG, Rg3, and PEG–GO–FA/ICG–Rg3, or co-treated with PEG–GO–FA/ICG–Rg3 and NIR laser. **(A,B)** Apoptosis analysis based on flow cytometry. **(C,D)** Western blot analysis of Bcl-2, Bax, caspase3, and cleaved caspase3 in the cells. Mean ± SD, **P < 0.01, ***P < 0.001.

Then, the effect of PEG–GO–FA/ICG–Rg3 on apoptosis of osteosarcoma cells was analyzed. Rg3 treatment induced the apoptosis of the MG63 and U2OS cells and PEG–GO–FA/ICG–Rg3 presented a higher impact (Figures 3A,B). Significantly, the co-treatment of PEG–GO–FA/ICG–Rg3 and NIR laser significantly improved the function of PEG–GO–FA/ICG–Rg3 in the induction of cell apoptosis in the MG63 and U2OS cells (Figures 3A,B). Similarly, the BAX and cleaved caspase3 expression were increased but Bcl-2 expression was decreased by Rg3 and PEG–GO–FA/ICG–Rg3, in which the co-treatment of NIR laser enhanced the effect of PEG–GO–FA/ICG–Rg3 on the phenotypes in the MG63 and U2OS cells (Figures 3C,D).

### The Effect of PEG–GO–FA/ICG–Rg3 on ROS and Autophagy of Osteosarcoma Cells

**FIGURE 4:**
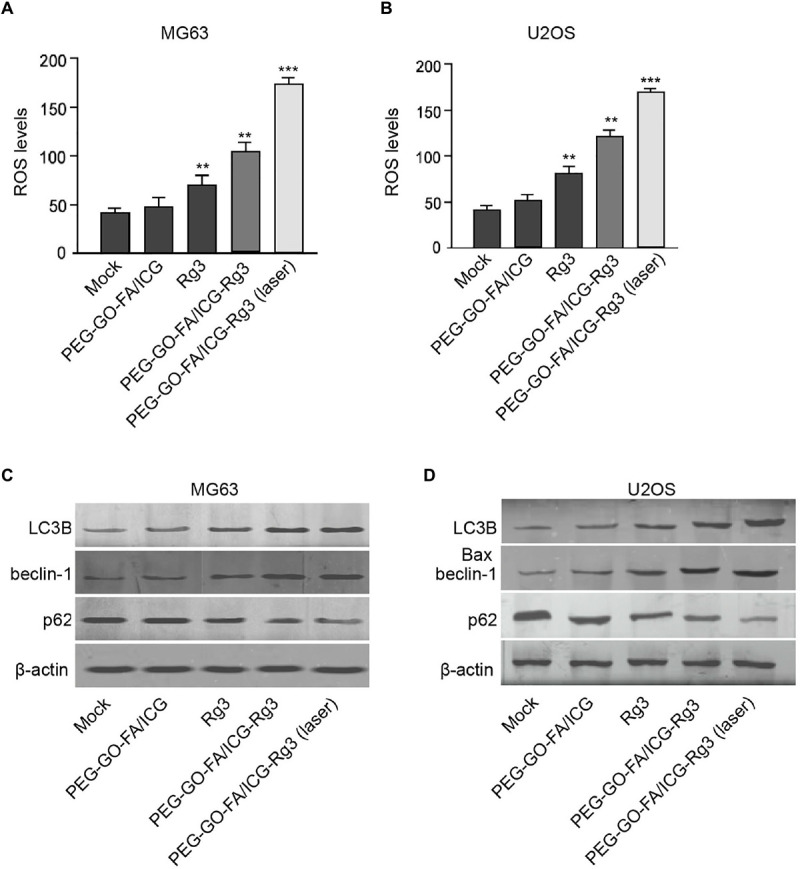
The effect of PEG–GO–FA/ICG–Rg3 on ROS and autophagy of osteosarcoma cells. **(A–D)** The MG63 and U2OS cells were treated with PEG–GO–FA/ICG, Rg3, PEG–GO–FA/ICG–Rg3, or co-treated with PEG–GO–FA/ICG–Rg3 and NIR laser. **(A,B)** ROS production analysis based on the diacetate (DCFH-DA) assays. **(C,D)** Western blot analysis of LC3B, beclin-1, and p62 in the cells. Mean ± SD, **P < 0.01, ***P < 0.001.

Next, we were interested in the effect of PEG–GO–FA/ICG–Rg3 on the autophagy and ROS production in the osteosarcoma cells. We observed that PEG–GO–FA/ICG–Rg3 enhanced Rg3-induced ROS production in the MG63 and U2OS cells, while the co-treatment of PEG–GO–FA/ICG–Rg3 and NIR laser significantly activated the PEG–GO–FA/ICG–Rg3-increased ROS levels in the cells (Figures 4A,B). Moreover, the LC3B and beclin-1 expression were enhanced but p62 expression was reduced by both Rg3 and PEG–GO–FA/ICG–Rg3 in the MG63 and U2OS cells, in which the treatment of NIR laser significantly stimulated the function of PEG–GO–FA/ICG–Rg3 in the cells (Figures 4C,D).

### The Effect of PEG–GO–FA/ICG–Rg3 on Stemness of Osteosarcoma Cell–Derived Cancer Stem Cells

**FIGURE 5:**
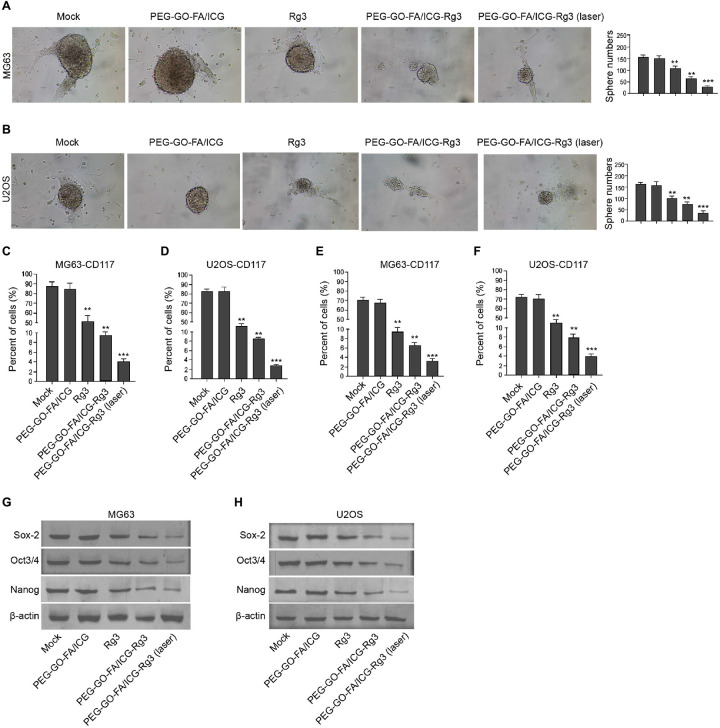
The effect of PEG–GO–FA/ICG–Rg3 on stemness of osteosarcoma cell–derived CSCs. **(A–H)** The MG63 and U2OS cells were treated with PEG–GO–FA/ICG, Rg3, and PEG–GO–FA/ICG–Rg3, or co-treated with PEG–GO–FA/ICG–Rg3 and NIR laser. **(A,B)** The sphere formation assays in the cells. **(C,D)** Flow cytometry analysis of CD117-positive cells. **(E,F)** Flow cytometry analysis of Stro-1-positive cells. **(G,H)** Western blot analysis of Sox2, Oct3/4, and Nanog in the cells. Mean ± SD, **P < 0.01, ***P < 0.001.

We tried to evaluate the stemness properties of osteosarcoma cells in the system. The treatment of PEG–GO–FA/ICG–Rg3 further reduced Rg3-inhibited sphere formation of the MG63 and U2OS cells, and the co-treatment of NIR laser significantly enhanced the effect of PEG–GO–FA/ICG–Rg3 on the sphere formation in the cells (Figures 5A,B). Meanwhile, the populations of CD117- and Stro-1-positive MG63 and U2OS cells were reduced by Rg3 and PEG–GO–FA/ICG could further inhibit this phenotype in the cells, in which the co-treatment of PEG–GO–FA/ICG–Rg3 and NIR laser remarkably stimulated the PEG–GO–FA/ICG–Rg3 function in the system (Figures 5C–F). Furthermore, the Sox2, Oct3/4, and Nanog expression was repressed by PEG–GO–FA/ICG–Rg3, in which NIR laser was able to significantly stimulate the inhibitory effect of PEG–GO–FA/ICG–Rg3 on the expression in the MG63 and U2OS cells (Figures 5G,H).

### The Effect of PEG–GO–FA/ICG–Rg3 on Tumorigenesis of Osteosarcoma Cells *in vivo*

**FIGURE 6:**
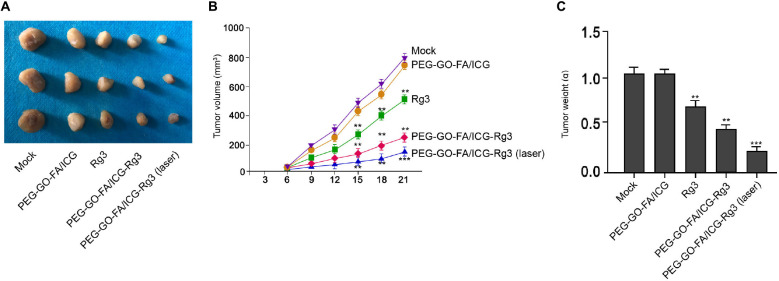
The effect of PEG–GO–FA/ICG–Rg3 on tumorigenesis of osteosarcoma cells *in vivo*. **(A–C)** Tumorigenicity assays in nude mice for five groups: control, PEG–GO–FA/ICG, Rg3, PEG–GO–FA/ICG–Rg3, PEG–GO–FA/ICG–Rg3 + NIR laser. **(A)** Representative images of dissected tumors from nude mice are presented. **(B)** The average tumor volume is calculated and shown. **(C)** The average tumor weight is calculated and shown. *N* = 5, mean ± SD, **P < 0.01, ***P < 0.001.

Next, we evaluated the combined effect of PEG–GO–FA/ICG–Rg3 and NIR laser on the tumorigenesis of osteosarcoma cells *in vivo.* Significantly, we found that the tumor growth of MG63 cells was suppressed by Rg3 and PEG–GO–FA/ICG–Rg3 could further repress this phenotype in the nude mice, in which the co-treatment of NIR laser remarkably stimulated the inhibitory effect of PEG–GO–FA/ICG–Rg3 on the tumor growth in the system (Figure 6A–C).

## Discussion

Osteosarcoma is a severe bone cancer with high mortality and poor prognosis. With the significant development of nanotechnology, the nanoparticle-based drug delivery system has been widely investigated in osteosarcoma development. Rg3 is a traditional Chinese natural compound that presents significant anti-cancer properties. In this study, we developed a PEG–GO–FA/ICG–Rg3 and identified that PEG–GO–FA/ICG–Rg3 improved PDT in osteosarcoma.

Previous studies have reported the application of nanoparticle-related drug delivery in the treatment of osteosarcoma. It has been reported that PEG–GO–FA/ICG nanoparticle-loaded MutT homolog 1 inhibitor enhances the chemo-photodynamic treatment effectiveness for osteosarcoma (Huang et al., 2020). Autophagy inhibitor increases ZnPc/BSA-based nanoparticle-induced photodynamic treatment through repressing the expression of PD-L1 in immunotherapy for osteosarcoma (Yu et al., 2019). AgBiS 2-nanoparticles combined with photodynamic treatment improved the phototherapy for malignant osteosarcoma (Cheng et al., 2020). Gold nanoparticles enhance photothermal/photodynamic treatment response for osteosarcoma (Xiong et al., 2021). Meanwhile, the anti-cancer activities of Rg3 in the development of osteosarcoma has been well recognized. Ginsenoside Rg3 enhances apoptosis and inhibits the proliferation of osteosarcoma cells (Li et al., 2018). Rg3 attenuates the metastasis and tumor growth of osteosarcoma by regulating EMT signaling and Wnt pathway (Mao et al., 2020). Rg3 stimulates DNA damage and induces apoptosis of osteosarcoma cells (Zhang et al., 2014). In addition, several investigations have reported that the nanoparticles can enhance the anti-cancer properties of Rg3. Targeting-drug delivery system based on polypeptide nanoparticles improves the anti-cancer effectiveness of Rg3 in colon cancer (Qiu et al., 2019). Nanoparticle-loaded Rg3 repressed metastasis and development of hepatocellular carcinoma (Ren et al., 2020). Our data showed that PEG–GO–FA/ICG–Rg3 repressed the proliferation, invasion, migration, and cancer cell stemness of osteosarcoma cells. PEG–GO–FA/ICG–Rg3 also induced apoptosis and autophagy in osteosarcoma cells. Also, NIR laser was able to significantly stimulate the anti-tumor activities of PEG–GO–FA/ICG–Rg3 *in vivo* and *in vitro*. Our data indicate an unreported and critical nanoparticle drug delivery system of Rg3 for the treatment of osteosarcoma. Meanwhile, there are some limitations in this study as well. The comparison of laser treatment only and the co-treatment with laser and PEG–GO–FA/ICG–Rg3 needs to be performed in future investigations. The tumorigenicity in nude mice was only observed to 21 days of injection and the effect of PEG–GO–FA/ICG–Rg3 on metastasis *in vivo* needed to be further investigated in a more appropriate model in the future. Also, more alternative methods are required in future investigations to analyze the function of PEG–GO–FA/ICG–Rg3 in osteosarcoma pathogenesis, such as initiation, ferroptosis, metastasis, and drug resistance. Moreover, the mechanism underlying the combination effect of PEG–GO–FA/ICG–Rg3 and PDT in stemness and malignant progression of osteosarcoma cells was not investigated in this study, which deserve to be explored in the future.

Therefore, we concluded that PEG–GO–FA/ICG–Rg3 improves PDT in inhibiting malignant progression and stemness of osteosarcoma cells. Our finding provides a promising and practical therapeutic strategy for the combined treatment of osteosarcoma.

## Data Availability Statement

The original contributions presented in the study are included in the article/**Supplementary Material**, further inquiries can be directed to the corresponding author.

## Ethics Statement

The animal study was reviewed and approved by Cangzhou Central Hospital.

## Author Contributions

S-LL and Y-HW designed and performed the experiments, analysed data, and wrote the manuscript. G-FL and LW designed and performed the experiments. YL, Z-YG, and CC designed the experiments, analysed data, and wrote the manuscript. All authors contributed to the article and approved the submitted version.

## Conflict of Interest

The authors declare that the research was conducted in the absence of any commercial or financial relationships that could be construed as a potential conflict of interest.
